# Histopathological Diagnosis of Eumycetoma With Paraspinal Sinuses: A Rare Case Report

**DOI:** 10.7759/cureus.29634

**Published:** 2022-09-26

**Authors:** Pankaj P Sonone, K. M. Hiwale

**Affiliations:** 1 Pathology, Jawaharlal Nehru Medical College, Datta Meghe Institute of Medical Sciences, Wardha, IND; 2 Pathology, Datta Meghe Institute of Medical Sciences, Wardha, IND

**Keywords:** madura foot, histopathology, eumycetoma, discharging sinuses, actinomycetoma

## Abstract

Mycetoma, well known as “Madura foot,” is a long-standing granulomatous infection of the skin and subcutaneous tissue. Causative organisms are filamentous fungi (eumycetes) and bacteria (actinomycetes). It characteristically presents with firm woody swelling, discharging sinuses with grains (containing the causative organism). Diagnosis in suspected cases can be made by microbiological evaluation, histopathological, cytological and radio imaging techniques. To differentiate between eumycetoma and actinomycetes histopathology is an accurate diagnostic modality as seen in the present case. We report a case of 42 years male who presented with swelling on his back with discharging sinus. Histopathological specimen containing multiple, irregular, greyish, whitish tissue pieces with skin attached all together measuring around 12×9×4 cm from the paraspinal region. The section shows histopathological features suggestive of eumycetoma. Periodic acid -Schiff stain showed the presence of septate, branching fungal hyphae and black granules. Eumycetoma can be accurately diagnosed by histopathological evaluation using a special stain. It is confirmatory and provides a guide for treatment plans with a high index of suspicion.

## Introduction

Mycetoma is a chronic granulomatous infection of the skin and subcutaneous tissue mainly involving extremities. It is a kind of implantation mycosis, which presents as large tumor-like swelling [1]. It can be caused by a taxonomically diverse microorganism, which includes bacteria (Actinomycetoma) and fungi (Eumycetoma) [2]. The most common causative agents include the fungus *Madurella mycetomatis* and the actinomycetes *Nocardia brasiliensis, Actinomadura madurae, Streptomyces somaliensis *and* Actinomadura pelletieri *[3].

World Health Organization has recently declared it a “neglected tropical disease” [4]. The cause for negligence is that it primarily affects low socioeconomic strata, residing in remote areas inaccessible to proper health care including a trained health worker, diagnostic tools and treatment. As the disease has a chronic course and poor response to treatment, it may add to the remark as a neglected tropical disease [4].

The accurate occurrence of mycetoma is not well known, but the maximum cases fall between the latitude of 15°S and 30°N, which is known as the “mycetoma belt.” The endemic countries consist of Sudan, Somalia, Senegal, India, Yemen, Mexico, and Venezuela [5]. Mycetoma commonly ‎involves the extremities, back, and gluteal region but any other part of the body can be affected.‎ It is slowly progressive with local tissue destruction. In advanced cases, bony destruction with functional impairment may happen [6]. The classical triad of Mycetoma comprises firm woody swelling, painless discharging sinuses, and presence of grains. Diagnosis can be done by various techniques involving clinical examination, grain examination, radio-imaging, histopathological evaluation, culture, and molecular diagnosis [7].

## Case presentation

**Figure 1:**
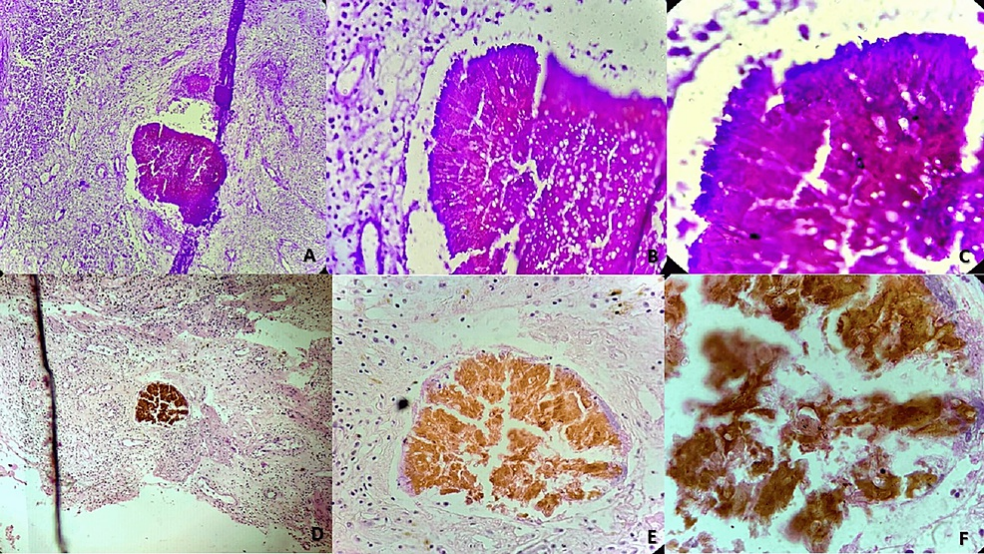
Histopathological appearance of eumycotic mycetoma (A) Periodic acid fast (PAS) staining of tissue showing broad septate hyphae of fungus on 10x magnification. (B) PAS staining 40x view. (C) PAS oil immersion view. (D) Hematoxylin and eosin (H&E) staining showing radiating sunburst appearance of septate hyphae surrounded by eosinophils, foreign body giant cells and other inflammatory cells on magnification of 10x. (E) H&E staining on 40x. (F) H&E 100x oil immersion view.

A 42-year-old male presented with a history of soft tissue swelling over the lumbosacral region for the past two years. The patient was a farmer by occupation. According to the patient, the swelling had gradually increased in size to become the current size over two years. But the reason for now seeking medical advice was the large size of the lesion restricting his mobility and affecting farming and daily activity. Associated trauma history is not recalled by the patient. First, it started with swelling over the lower side of the back, then multiple discharging sinuses with granules over a period of two years. As it was huge in size, surgical resection was advised. On gross examination, it was approximately 12×9×4 cm in size and had multiple, irregular, greyish, whitish tissue pieces with skin attached all together. Periodic acid-fast (PAS) staining of tissue has shown branched septate hyphae of fungus (Figures 1A-1C). On hematoxylin and eosin staining (H&E) radiating sunburst appearance of septate hyphae surrounded by eosinophils, foreign body giant cells, and other inflammatory cells was seen (Figures 1D-1F).

## Discussion

Mycetoma is a rare pathology caused by either a filamentous fungus (eumycetoma) or by filamentous bacteria (Actinomycetes species) [2]. Mycetoma is characteristically found in agronomic workers who generally walk barefoot in dry, dusty, and humid conditions. Trivial injury can lead to the entry of the pathogen into the skin from soil [8].

The grains discharged from sinuses differ in size, color, and consistency. These topographies can be utilized for quick provisional identification of causative agents [9]. But grains of many species have overlying structural features so culture can be done for definitive identification of causative factors.

The incubation period of these organisms fluctuates from several weeks to months [9]. Development of sinuses usually occurs only after 6-12 months. In untreated cases involvement of underlying fascia, muscle and bone is common. Sometimes dissemination through regional lymph nodes may happen [10]. Actinomycetic mycetomas are known for the faster and more invasive course with more sinuses than eumycotic variants [9].

The key differential diagnoses include chronic bacterial osteomyelitis, tuberculosis, and Buruli ulcer. Other deep fungal infections such as blastomycosis or coccidioidomycosis and leishmaniasis, yaws, and syphilis should be considered [11]. Identification of the causative bacteria/fungi is often difficult using conventional microbiological techniques. Culture and sensitivity are useful but time-taking processes. Tissue or bone biopsy can be better evaluated by molecular (DNA sequencing method) if available. Histopathological evaluation with clinical correlation aids in confirmatory diagnosis.

## Conclusions

As this disease is generally found in low socio-economic strata in developing countries, where facilities for molecular diagnosis are inaccessible to common people. As well, due to the chronic painless nature of the disease, presentation is very late and surgical resection with antibiotic and antifungal coverage will become a mainstay of treatment. In this situation, the histopathological diagnosis will be a good diagnostic modality to differentiate between its fungal or bacterial origin and also for a better outcome for the patient. The unusual location of mycetoma should be considered along with keeping in mind the probability of another differential diagnosis of mycetoma especially tuberculosis which is more prevalent in developing countries.
